# The prevalence of metabolic syndrome in patients receiving antipsychotics in Qatar: a cross sectional comparative study

**DOI:** 10.1186/s12888-018-1662-6

**Published:** 2018-03-27

**Authors:** Samer Hammoudeh, Suhaila Ghuloum, Ziyad Mahfoud, Arij Yehya, Abdulmoneim Abdulhakam, Azza Al-Mujalli, Mahmoud Al-Zirie, Mohamed Osman Abdel Rahman, Angela Godwin, Noura Younes, Yahya Hani, Dennis Mook-Kanamori, Marjonneke Mook-Kanamori, Reem El Sherbiny, Hassen Al-Amin

**Affiliations:** 1 Department of Research, Weill Cornell Medicine – Qatar, Doha, Qatar; 2 Department of Psychiatry, Rumailah Hospital, Hamad Medical Corporation, Doha, Qatar; 3 Department of Health Policy and Research, Weill Cornell Medicine – Qatar, Doha, Qatar; 4 Primary Health Care Corporation, Doha, Qatar; 5 Endocrinology Department, Hamad General Hospital, Doha, Qatar; 6 Department of Laboratory Medicine and Pathology, Hamad Medical Corporation, Doha, Qatar; 7 Department of Physiology and Biophysics, Weill Cornell Medicine – Qatar, Doha, Qatar; 8 Department of Psychiatry, Weill Cornell Medicine – Qatar, Education city, P.O. Box 24144, Doha, Qatar

**Keywords:** Metabolic syndrome, Antipsychotics, Arabs, Mental illness

## Abstract

**Background:**

Metabolic abnormalities are common in patients maintained on antipsychotics. These abnormalities increase the risk of cardiovascular diseases and mortality in this population. The aim of this study is to assess the prevalence of metabolic syndrome (MetS) in subjects maintained on antipsychotics relative to controls in Qatar, and to assess the factors contributing to the development of MetS.

**Methods:**

A cross sectional design was used to collect data and fasting blood samples from subjects maintained on antipsychotics for at least six months (*n* = 112) and from a control group (*n* = 114). The groups were compared in regard to prevalence of MetS, and multiple regression analysis was used to determine the risk factors in each group.

**Results:**

The two groups (antipsychotics vs. control) were similar in regard to age (35.73 ± 10.28 vs. 35.73 ± 8.16 years) and gender ratio. The MetS was higher among the subjects on antipsychotics, but this difference did not reach statistical significance. Blood pressure (BP) was significantly higher in the antipsychotics group and BMI was the major risk factor to develop MetS in this group.

**Conclusions:**

The prevalence of MetS in both groups is high and mostly attributed to obesity and high BP. Public health interventions are needed to address this major health problem overall. Larger studies are needed to further assess the impact of antipsychotics and mental illness on the development of MetS.

## Background

Metabolic syndrome (MetS) comprises specific risk factors for cardiovascular disease. These factors are increased waist circumference, raised fasting glucose, hypertension, and dyslipidemia. Several organizations have established criteria for the identification of MetS such as the National Cholesterol Education Program/Adult Treatment Panel III (ATP III) or International Diabetes Federation (IDF) [1]. Chronic mental disorders like schizophrenia and bipolar disorder have been associated with higher prevalence of MetS [2, 3], which is contributing to the increased rates of mortality and morbidity in these patients [2]. Antipsychotics are also known to cause weight gain and increase in glucose or cholesterol levels [4–6], which also increase the prevalence of MetS.

Both groups of antipsychotics, first generation antipsychotics (FGA) and second generation antipsychotics (SGA), are indicated for the treatment of schizophrenia [7]. Many SGA are also indicated for bipolar disorder [8] and as augmentation for resistant depression [9]. The metabolic side effects are more frequently seen with SGA than with FGA [10]. Several studies have shown that SGA have differential risk on weight gain and MetS with clozapine and olanzapine demonstrating the highest risk while ziprasidone and aripiprazole have the lowest [11, 12]. However, other studies from Japan [13], Venezuela [14], and Italy [15] did not show that antipsychotics increase the risk of MetS compared to control subjects. Bajaj et al. (2013) showed a positive correlation between the duration of treatment and the prevalence of MetS among those taking SGA [16]. In addition, Gautam and Meena (2011) reported that 11.66% of the patients with schizophrenia developed MetS after four months of being on antipsychotics [17]. Thus, it seems that there are racial and cultural differences when studying the prevalence of MetS in patients with schizophrenia and maintained on antipsychotics.

In the Arab countries, several studies have suggested higher prevalence of MetS in these populations [18–21]. For example, in Qatar, Bener et al. [22] reported a MetS prevalence rate among the general population of 26.2% and 36.9% based on ATP III and IDF criteria, respectively. A cross sectional study, which looked into obese subjects attending primary health care centers in Qatar showed an overall prevalence rate of 46.3% (IDF criteria) [23]. The few prevalence studies conducted in the Arab countries on mental illness and MetS have shown similar results but mostly these were either retrospective starting with the patients with mental illness or without a control group. A Lebanese study reported a prevalence rate of MetS among schizophrenia patients on SGA of 32.3% (ATP III criteria) and of 48.4% (IDF criteria) [24]. A Kuwaiti study on schizophrenia inpatients showed prevalence rates of 18.8%, 23.2%, and 24.9% according to the ATP III, American Heart Association/National Heart, Lung, and Blood Institute (AHA/ATP III-A), and IDF definitions, respectively [25]. Severity and duration of illness, along with age, were reported as factors that correlate with increased prevalence of MetS [25]. Similarly, a cross sectional study reported a MetS prevalence rate of 43.6% among schizophrenia Palestinian patients according to the ATP III definition [26]. A cross sectional study in the United Arab Emirates (UAE) reported MetS (ATP III criteria) prevalence rates of 58.9%, 44.2% and 34.2% among patients with schizophrenia, bipolar disorder and major depression, respectively. The same study reported the following as factors that are more common in patients with MetS: advanced age, obesity, female gender, history and/or family history of diabetes, longer duration of mental illness and of psychotropic treatment [27]. None of the above studies had a control group to judge if the high prevalence of MetS is different from that in the general population to conclude that the mental illness and the psychotropics are implicated in this high prevalence of MetS. One previous study from Qatar assessed patients with schizophrenia and showed a significantly higher prevalence of MetS (ATP III criteria) when compared to control subjects not receiving antipsychotics and with no mental illness (36.5% vs. 18.7%) [28]. Central obesity was the most commonly encountered risk factor among patients with schizophrenia compared to controls (63.9% vs. 45.7%) [28]. This study also included schizophrenia patients only, and thus few studies in the Arab countries have assessed directly the association of antipsychotics with MetS.

The aim of this cross-sectional study in Qatar was to compare the prevalence of Mets in subjects maintained on antipsychotics for at least six months vs. controls with no mental illness and not taking antipsychotics. We hypothesized that those taking antipsychotics will have higher prevalence of MetS. A secondary analysis was done to determine the factors contributing to having MetS in our sample.

## Methods

### Setting and participants

**Fig. 1:**
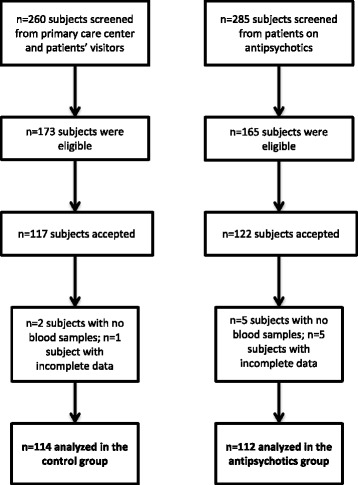
Flow chart showing the number of subjects screened, eligible, completed the assessments and the final number used in the analysis

Qatar is a rapidly growing country where Qataris and other Arabs are about 25% of the general population while the others are expatriates from many different countries especially from south Asia. A total of 226 participants were recruited between December 2012 and June 2014. Patients maintained on antipsychotics (*n* = 112) were recruited from the Psychiatry Department at Rumailah hospital, Doha, Qatar. This department is the only psychiatric facility in Qatar. The control subjects (*n* = 114) were recruited from the Primary Health Care Center, Doha, Qatar and the patients’ visitors at the Department of Psychiatry. The treatment teams at both facilities screened the subjects using convenience sampling and invited those who were eligible according to the inclusion and exclusion criteria presented below. About 74% of the eligible subjects for the antipsychotics group agreed to participate and about 68% from those eligible for the control group were enrolled (see the flow chart in Fig. 1).

The inclusion criteria for patients on antipsychotics were: (a) 18–65 years of age, (b) have been taking antipsychotics for at least the last six months, (c) not known to have MetS and (d) able to sign a consent form. The exclusion criteria were: (a) have been taking Lithium, Nortriptyline or Amitriptyline for the past three months as these medications are associated with significant weight gain and (b) have current substance abuse or dependency disorder. The psychiatric diagnosis of these subjects was obtained from the patients’ records and relied on the Diagnostic and Statistical Manual of Mental Disorders (DSM-IV) criteria at that time. The inclusion and exclusion criteria for the control group were the same as the antipsychotics group except that subjects had not received any antipsychotics. The control subjects were screened using the Mini International Neuropsychiatric Interview (MINI-6) to make sure that they have no psychiatric disorder. Approval for the study was obtained from Hamad Medical Corporation (HMC) and Weill Cornell Medicine-Qatar (WCM-Q) Institutional Review Boards. All participants signed a written informed consent after they received clear information about the study and the duration of procedures.

### Sample size calculation

With 75 cases (patients receiving antipsychotics) and 75 controls (general population) matched for age and gender, we expected to detect a difference of 26% in the prevalence of MetS between cases and controls, with a power of 90% and a significance level of 5%. These calculations were done using the proportion of MetS of 26% among the general population [29] and postulating a two-fold increase in the rate of MetS among patients taking antipsychotics as compared to the general population [30]. As the difference in the prevalence might not be as large as in other populations and to allow for subgroup analyses we elected to recruit 110 patients and 110 controls. This will allow us to detect a smaller difference of about 20% between the two groups. The numbers recruited are slightly higher as we had some incomplete data on few subjects.

### Diagnosis of MetS

Two diagnostic criteria for MetS were used in this study. The ATP III criteria [31] and the IDF criteria [32], which are shown in Table 1.

**Table 1:** Summary of the ATP III and IDF criteria for MetS

	ATP III (3 of 5)	IDF (2 of 4, central obesity is mandatory)
Waist circumference	Abdominal obesity, based on waist circumference > 102 cm for men, and > 88 cm for women	Central obesity based on ethnicity specific values^a^ (for Europids: waist circumference ≥ 94 cm for men, and ≥ 80 cm for women).
Triglycerides	≥ 150 mg/dL	≥ 150 mg/dL, or specific treatment for this lipid abnormality.
HDL cholesterol	< 40 mg/dL for men < 50 mg/dL for women	< 40 mg/dL for men, < 50 mg/dL for women, or specific treatment for this lipid abnormality.
Blood pressure (BP)	≥ 130/ ≥85 mmHg	Systolic BP ≥ 130 mmHg, or diastolic BP ≥ 85 mmHg, or treatment of previously diagnosed hypertension.
Fasting glucose	≥ 110 mg/dL	≥ 100 mg/dL, or previously diagnosed type 2 diabetes mellitus.

^a^European measures were used due to the lack of regional standards. ATP III: Adult Treatment Panel III. IDF: International Diabetes Federation. MetS: Metabolic syndrome. HDL: High-density lipoproteins

### Procedures and measurements

The questionnaires covered the following categories: sociodemographic data, medical/psychiatric history, family history and type/duration of antipsychotics intake. The vital signs (blood pressure and heart rate using General Electric Critikon Dinamap Pro 400 V2) and anthropometric measurements (height, weight, waist and hip circumference using Seca instruments) were obtained from all participants. The waist circumference was measured at the level midway between the lowest rib and the iliac crest.

Four raters were trained to administer these validated questionnaires and measures according to specific standardized operating procedures. The raters applied the same procedures to both the active and control subjects within 1–2 days after obtaining the informed consent. The subjects were then asked to be present, within 1–2 days, to draw blood samples after fasting for 10–12 h. All the blood tests (CBC, lipid profile, HbA1c, fasting glucose, liver and kidney function tests) were done according to the same standardized procedures of the laboratories of Rumailah hospital, Doha, Qatar.

### Statistical analysis

All statistical analyses were conducted using the Statistical Product and Service Solutions (SPSS) version 23. The level of significance was set at 0.05 level. Descriptives were reported using frequency (percentage) for categorical variables and mean ± SD (or interquartile range) for continuous measures respectively. To compare groups, chi square test was used to analyze categorical variables and independent two-tailed t-test was used with continuous variables. Bivariate analysis (antipsychotics vs. control) was conducted to compare the sociodemographic and clinical characteristics of the two groups. We also did bivariate analysis for each group separately looking at the differences between those with MetS and those without (MetS vs. no MetS). The Kolmogorov-Smirnov test was used to assess normality. In this regard, Mann-Whitney U was used. All bivariate analyses were done with Bonferroni corrections for multiple comparisons. In order to assess the factors that are contributing to having MetS in each of the groups (antipsychotics or controls), multiple logistic regression (backward conditional, as this is an exploratory approach) was done, separately for each group, using the significant variables from the bivariate analysis (MetS vs. no MetS). The best-fit model was tested using Hosmer and Lemeshow test and by checking Cox and Snell R^2^ and Nagelkerke R^2^.

## Results

### Sociodemographic and medical characteristics

There were no significant differences between the two groups in regard to age or gender (Table 2). When compared to the control group, the patients on antipsychotics had significantly higher number of Qataris/Arabs, single individuals, and unemployed participants with school education only. In the control group the significant majority were Non-Arabs, married, never smoked, college graduates and employed (Table 2). The Arab population included the following countries: Algeria, Egypt, Jordan, Kuwait, Lebanon, Libya, Morocco, Palestine territories, Qatar, Kingdom of Saudi Arabia, Somalia, Sudan, Syria, Tunisia and Yemen. The Non-Arabs were mostly from south Asia (Bangladesh, India, Nepal, Pakistan, and Sri Lanka) and sporadic cases from France, Iran, Nigeria, Kenya, Tanzania, Philippines and United States. In regard to medical history there were significantly more smokers in the antipsychotics group. Furthermore, there were no significant differences between the two groups in the blood count measures, liver, kidney and thyroid function tests.

**Table 2:** Sociodemographic and medical characteristics of study participants by group

Variables	Antipsychotics group (*n* = 112)	Control group (*n* = 114)
Age *(mean ± SD)*	35.73 ± 10.28	35.73 ± 8.16
Gender *n (%)*		
Male Female	73 (65.2%) 39 (34.8%)	69 (60.5%) 45 (39.5%)
Ethnicity *n (%)*		
Qataris and Arabs Non-Arabs	79 (71.2%)* 32 (28.8%)	52 (45.6%) 62 (54.4%)**
Marital status *n (%)*		
Married Single Divorced	43 (38.4%) 57 (50.9%)* 12 (10.7%)*	83 (72.8%)** 30 (26.3%) 1 (0.9%)
Degree *n (%)*		
None School Vocational College/graduate	4 (3.6%) 72 (64.3%)* 7 (6.3%) 28 (25.0%)	4 (3.5%) 45 (39.5%) 15 (13.2%) 49 (43.0%)**
Employment *n (%)*		
Employed Not employed Other (student, retired, etc.)	41 (36.6%) 62 (55.4%)* 9 (8%)	103 (90.4%)** 9 (7.8%) 2 (1.8%)
Smoking/current *n (%)*		
Yes No	40 (36.7%)* 69 (63.3%)	18 (18%) 82 (82%)**
Smoking/ever *n (%)*		
Yes No	52 (47.3%)* 58 (52.7%)	26 (22.8%) 88 (77.2%)**
History of Diabetes *n (%)*		
Yes No	9 (8.2%) 101 (91.8%)	8 (7.3%) 101 (92.7%)
Family history of diabetes *n (%)*		
Yes No	74 (66.1%) 38 (33.9%)	74 (64.9%) 40 (35.1%)
Body mass index (BMI) (mean ± SD)	29.12 ± 6.95	27.74 ± 5.05

*Higher than the control group; **Higher than the antipsychotics group (*p* < 0.05)

### Psychiatric profile of the antipsychotics group

Patients who were on antipsychotics had mainly the following three diagnoses: bipolar (42.0%), schizophrenia/schizoaffective (35.7%), and depression (7.1%). The remaining 15.2% included the following disorders: personality, obsessive compulsive, delusional, psychotic not otherwise specified or mood not otherwise specified.

The majority had history of multiple hospitalizations and were on SGA, where 43 subjects (38.4%) were on olanzapine, 36 on risperidone (32.1%), 17 on aripiprazole (15.2%), 12 on quetiapine (10.7%), 6 on paliperidone (5.4%), and 3 on clozapine (2.7%). In regard to FGA, 3 were on flupenthixol (2.7%), 4 on trifluoperazine (3.6%), 15 on haloperidol (13.4%), and 16 on chlorpromazine (14.3%). It is worth noting that 21 patients were on a combination of FGA and SGA, and few others were receiving more than two antipsychotics. As many patients received multiple antipsychotics throughout the duration of their illness we calculated also the mean duration of the last antipsychotic received, which was over a year (Table 3).

**Table 3:** Clinical characteristics of patients maintained on antipsychotics

Antipsychotics group (*n* = 112)	Frequency	Percentage
Type of antipsychotic		
FGA SGA Combination of both	15 76 21	13.4% 67.9% 18.8%
Hospitalized before		
Yes No	82 30	73.2% 26.8%
Suicide attempt		
Yes No	26 80	23.2% 71.4%
	Mean	SD
Duration of illness *(years)*	12.6	9.37
Number of Hospitalizations	4.12	4.02
Total psychotropic medications taken/lifetime	4.36	2.81
	Mean	IQR^a^
Dose of antipsychotics^b^	689.52	405.39
Duration of last psychotropic medication *(months)*	15.86	12

^a^IQR: Interquartile range, ^b^Chlorpromazine 100 mg/day equivalent doses for antipsychotics [33]

### Prevalence of MetS

**Fig. 2:**
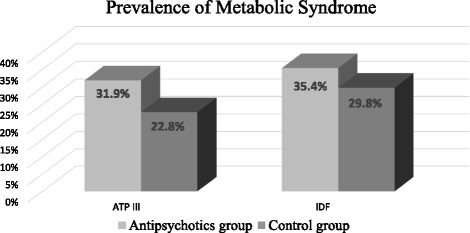
Metabolic syndrome (MetS) prevalence rates among participants based on the ATP III and IDF criteria. Patients with mental illness who were maintained on antipsychotics (*n* = 112) for at least 6 months were compared to a control group (*n* = 114). No significant difference in the prevalence of MetS between the two groups. ATP III: Adult Treatment Panel III. IDF: International Diabetes Federation

The prevalence of MetS was higher in the antipsychotics group (31.9% for ATP III and 35.4% for IDF criteria) when compared to the control group (22.8% for ATP III and 29.8% for IDF criteria) (Fig. 2). However, this increase did not reach statistical significance, *p* = 0.12 for ATP III and *p* = 0.37 for IDF.

A comparison between the groups on the different metabolic factors is shown in Table 4. Systolic (*p* = 0.001) and diastolic (*p* < 0.001) blood pressures (BP) were significantly higher in the antipsychotics group.

**Table 4:** Distribution of MetS factors (Mean ± SD) by group

MetS factor	Antipsychotics group (*n* = 112)	Control group (*n* = 114)
Triglycerides *(mg/dL)*	1.60 ± 1.05	1.54 ± 0.99
HDL *(mg/dL)*	1.22 ± 0.32	1.20 ± 0.44
Fasting plasma glucose *(mg/dL)*	6.28 ± 4.94	5.72 ± 2.16
Systolic BP *(mmHg)*	127.69 ± 13.70*	120.87 ± 15.22
Diastolic BP *(mmHg)*	81.29 ± 10.75*	75.49 ± 12.40
Waist circumference *(cm)*	97.16 ± 14.09	99.03 ± 92.29

*Higher than the control group (*p* < 0.05). HDL: High-density lipoproteins

### Biopsychosocial profile and MetS

Bivariate analysis of the sociodemographic and clinical characteristics in the antipsychotics group alone showed that participants with MetS (vs. no MetS) had longer duration of illness (*p* < 0.05, ATP and IDF) and increased duration of treatment with the last antipsychotic (*p* < 0.05, ATP); they were also older (age, *p* < 0.05, ATP), mostly Arabs (χ^2^ = 3.91, *p* = 0.048, IDF), obese (BMI, *p* < 0.05, ATP and IDF), known to have diabetes (χ^2^ = 5.49, *p* = 0.02, IDF) and current smokers (χ^2^ = 4.10, *p* = 0.04, IDF). There were no significant differences in regard to gender, marital status, diagnosis, type, number or equivalent dose of antipsychotics. The same analysis in the control group showed that the following factors were significantly more common in the subjects with MetS: older age (*p* < 0.05, ATP and IDF), being male (gender, χ^2^ = 5.16, *p* = 0.02, IDF), non-Arab (ethnicity, χ^2^ = 7.16, *p* = 0.007), obesity (BMI, *p* < 0.05, IDF), having diabetes (χ^2^ = 4.33, *p* = 0.04, ATP) and current smokers (χ^2^ = 4.43, *p* = 0.04, ATP). The other demographic and medical features were not significant.

### Multiple regression analysis and MetS

A multiple logistic regression (backward conditional) was carried out for each group separately using the significant factors from the above bivariate analyses to assess what factors are independently contributing to the presence of MetS (as per IDF criteria that gave more people with MetS) in each group. The predictors in the control group were age, gender, ethnicity, BMI, history of diabetes and currently smoking. The same variables were added in the analysis of the antipsychotics group in addition to the duration of psychiatric illness and duration of last antipsychotic treatment. We elected to add also the current dose of antipsychotics, as it is known to contribute to the metabolic effects.

In the control group the full regression model with all the six predictors was significant (χ^2^ = 26.95, df = 6, *p* < 0.001). The results of Cox and Snell R^2^, and Nagelkerke R^2^ showed that the model explained between 25% and 35% of the variance predicted by these independent factors. This best-fit model was ascertained by the Hosmer and Lemeshow test (χ^2^ = 3.44, df = 8, *p* = 0.91). The whole model gave an overall 77% correct rate of the outcome (MetS vs. no MetS). The predictors that remained significant after controlling for the other ones were: age (odds ratio: OR = 1.087, 95% confidence interval (CI): 1.015–1.363, *p* = 0.017), being non-Arab (OR = 9.693, 95% CI: 2.427–38.713, *p* = 0.001) and BMI (OR = 1.197, 95% CI: 1.054–1.359, *p* = 0.006).

In the antipsychotics group the full regression model was significant (χ^2^ = 17.85, df = 9, *p* < 0.037). The results of Cox and Snell R^2^, and Nagelkerke R^2^ showed that the model explained between 27% and 37% of the variance predicted by these independent variables. The Hosmer and Lemeshow test confirmed that this model is a good fit (χ^2^ = 8,57, df = 8, *p* = 0.38). The overall success rate in this model was also 77%. However, the only predictor that remained significant was the BMI (OR = 1.162, 95% CI: 1.047–1.289, *p* = 0.005).

## Discussion

The aim of this cross-sectional study was to check the metabolic effects of antipsychotics when compared to a control group. The prevalence of MetS was not significantly different between the two groups but the systolic and diastolic BPs were significantly higher in the group receiving antipsychotics. However, a difference of 9% (ATP III) or 5% (IDF) in the prevalence rates of MetS is clinically relevant, but probably did not reach statistical significance because of the high prevalence of MetS in the general population in our study compared to other population studies [14, 19, 21]. In regard to the MetS prevalence in patients with mental illness, our rates are very close to the ones reported previously from Qatar [28], where the overall prevalence of MetS in schizophrenia patients was 36.5% vs. 31.9% (IDF criteria) in our study. However, in the same study the prevalence of MetS in the control group was 18.7%, which is very different from the prevalence reported in a population study from Qatar by the same group where the prevalence of MetS in the general population was 26.5% [29]. The prevalence rate of MetS in the antipsychotic group in our study (31.9%) is higher than rates reported on the Kuwaiti (18.8%) [25], but lower than the rates reported from UAE (58.9%) [27] and Palestinian patients with schizophrenia (43.6%) [26]. It is worth mentioning that these studies were basically on patients with specific diagnoses without taking into consideration the type of medications received and did not have a control group to compare with. As presented in the introduction [13, 14, 30] it seems that there are also differences among nations in the prevalence of MetS in the general population and in the degree of worsening of these metabolic factors with antipsychotics.

The high level of obesity among the general population in our study is probably what contributed to the high prevalence of MetS in our sample, as previous studies from the Arabic Gulf region [33] and from Qatar [34] showed high obesity rates. This is also supported by our results on multiple regression analyses where high BMI was a major risk factor to develop MetS in both groups. This also explains the difference in the prevalence rates of MetS based on the two different criteria used in this study, as the IDF definition mandates having central obesity to confirm MetS, in comparison to the ATP III criteria that do not.

Our results also showed significant differences in BP, when comparing the antipsychotics vs. control group. Hypertension is also known to be very common in the population of Qatar with a prevalence rate of 37.1% [34]. Although antipsychotics are known to cause mild orthostatic hypotension, the increased BP in patients on antipsychotics could be secondary to the increased weight gain as they had also higher BMI. Other studies using logistic regression models have shown that obesity is associated with hypertension as well as dyslipidemia [35]. Few case studies have reported increased BP with few antipsychotics [36, 37]. Still others suggested that maintenance on antipsychotics could lead to autonomic dysfunction (and increased adrenergic activity) and thus higher BP and more metabolic and cardiovascular effects [38].

When participants were compared based on the presence of MetS, several factors emerged as possibly contributing to the high prevalence of MetS in the general population. These factors are older age, being male, expatriate, smoker, having diabetes and obesity. Our regression analysis from the controls confirmed older age and higher BMI as risk factors for developing MetS in the general population of Qatar [29, 39] as have been shown in other countries [20, 21]. The former studies from Qatar also showed that females are more at risk to have MetS, but in our study gender was not a risk factor, probably because the majority of our sample were males. It is worth noting that the majority of the population in Qatar is men (75%) as many workers migrate to Qatar without their families. However, the studies from other countries reported mixed results in regard to gender [21, 35, 40]. Furthermore, our study also showed that being of non-Arab origin was a risk factor for developing MetS. The majority of non-Arabs were from south Asia but the numbers from the different countries were small to warrant statistical comparison. It is worth noting that in our sample there were more Arabs in the antipsychotics group while in the control group the majority were non-Arabs, which could explain this variability between the two groups. Still ethnicity/nationality showed significant variations in the prevalence of MetS in patients with mental illness and maintained on antipsychotics [5, 13, 14, 24–27].

In addition, for the group on antipsychotics, longer duration of illness and of maintenance on current antipsychotics are probably also contributing to the presence of MetS in the patients on antipsychotics. In this group, regression analysis showed obesity as a risk factor to develop MetS. A study on Han Chinese patients with schizophrenia had also demonstrated high BMI as an independent risk factor to develop MetS [40]. Similar findings were reported in a prospective study on patients with bipolar disorder from Italy where older age, higher BMI and exposure to antipsychotics were independently associated with the risk of developing MetS after two years [41]. The study on Palestinian patients with schizophrenia showed also higher prevalence of MetS in patients who are older, with abdominal obesity, longer duration of illness, smoking and with high BP, except for being more prevalent in females in their study. However, none of these sociodemographic features were significant in regression analysis [26]. Contrary to the general belief that SGA are more associated with MetS [11, 12], others have shown that polypharmacy and type of antipsychotics were not associated with increased risk of MetS in patients maintained on antipsychotics [42, 43]. Our results did not find any significant differences between those with MetS vs. not when comparing the type of antipsychotics and equivalent doses of antipsychotics.

This prevalence study has several strengths like the cross-sectional design, having a control group with similar protocols of assessment, trained research staff and the focus on the effects of antipsychotics. However, there are limitations that might limit the interpretation of findings like the sample size, the preponderance of males in the sample recruited, the unequal representation of the different nationalities and the large variability in the duration of illness and dosage of medications, as these factors can affect the severity of the metabolic effects of antipsychotics and should be controlled for in future studies.

## Conclusions

Our results showed that the prevalence of MetS is high in both the control group and the patients on antipsychotics in Qatar. The major risk factor and contributor to this increase is the high prevalence of obesity in this region. BP was the only constituent of MetS that was significantly higher in patients maintained on antipsychotics. Both groups can benefit from interventions to reduce weight gain and control high BP especially with longer duration of illness and maintenance on antipsychotics.

## Abbreviations

AHA: American Heart Association
ATP III: Adult Treatment Panel III
BMI: Body Mass Index
BP: Blood Pressure
DSM IV: Diagnostic and Statistical Manual of Mental Disorders, version four
FGA: First Generation Antipsychotics
HDL: High-density lipoproteins
HMC: Hamad Medical Corporation
IDF: International Diabetes Federation;
IQR: Interquartile Range
MetS: Metabolic Syndrome
MINI: Mini International Neuropsychiatric Interview
QNRF: Qatar National Research Fund
SGA: Second Generation Antipsychotics
SPSS: Statistical Product and Service Solutions
UAE: United Arab Emirates
WCM-Q: Weill Cornell Medicine-Qatar
